# Influenza Personally Conducted

**Published:** 1890-02-15

**Authors:** 


### THE DOCTOR'S PULPIT.

### INFLUENZA PERSONALLY CONDUCTED.

"A little more vitality to-day, a little more steadiness in the legs, a little more courage about the heart, a little more brightness in the brain." Thus, at the end of three weeks, did a victim of influenza, followed by bronchitis caused by too early exposure, record his personal experiences. Whatever epidemic influenza may be in its origin and essence, it is a disease that produces very marked effects upon some persons. Many medical men in this country, the writer included, were at first inclined to regard the malady as the product of a foreign "fad," coupled with a newspaper "craze." It is to be hoped that most of those medical sceptics have had a sharp taste of the visitor's quality; not, of course, by way of punishment for their scepticism, but by way of instruction, and in the interests of their patients.

It seems a little late in the day, seeing that the epidemic is subsiding, to record personal experiences. Such experiences may, however, serve the double purpose of interesting those who, having had the disease, can compare notes; and of offering some hints whereby those who have hitherto escaped may be enabled to continue their self-protection to a finally successful issue.

What is the real cause of the malady? A specific microbe, or an atmosphere persistently chilly and damp? A brief sketch of the commencement and after history of one well-marked case may help to answer this important and fundamental question. On Monday, January 13th, the particular person here considered travelled to Brighton in the afternoon; and although he was warmly clothed and made the journey in a Pullman car, in which the thermometer recorded a temperature of 69 degrees Fahrenheit, he felt decidedly chilly all the way down. Brighton itself on his arrival was enshrouded in a dense white sea fog, which chilled to the marrow. The house visited had an influenza case in it, and the subject of this little history stayed all night there. On going to bed at ten o'clock, although there was a good fire in the room, and the sleeping accommodation consisted of an old-fashioned feather bed, with abundance of blankets and an eider-down, the shivering decidedly increased, and was accompanied by marked prostration for at least two hours. Then followed what, for that particular person, was a very unusual thing—severe, continuous, and extremely painful cramps in the right leg. These prevented sleep until about two in the morning, and were present in a modified form until breakfast time. Strange to say, the patient felt almost well all the next day. So much was this the case that he considered he had just touched the edge of the epidemic but had escaped its virulence. On Tuesday evening, feeling well, he went to a lecture and sat for about two hours in a hall warmed apparently only by gaslight. Towards the end of the lecture the chills began to return; nevertheless, he went to bed at the ordinary time, slept all night, and waked in the morning apparently almost as well as usual. But at mid-day a different set of symptoms began to show themselves. An overpowering prostration came on, almost quite suddenly, accompanied by severe frontal headaches. The temperature rose, and aching pains, with cramps, were felt in the back, arms, legs, chest, abdomen, neck, and all over. The fever steadily increased in spite of remedies, until bedtime. The thirst was great, the prostration extreme, and the feeling of illness and collapse indescribable. The patient felt as if he must die, and but for the inconvenience of such an event to his family, imagined that he would be very glad to do so.

This fully developed fever and prostration may be considered to have marked the close of the first stage of the attack. There was no sleep that night; but thirst, overpowering langour, an irresistible desire for stimulants, and a longing for the break of day. When morning came the appetite was found to be completely abolished. Solid, and even liquid, food excited loathing, and the former could not be taken at all. A sense of duty compelled the unwilling victim to swallow liquids, and he took at intervals of one or two hours beef tea, strong soup, egg flip, cocoa, champagne, whiskey and water, sal volatile, and so on. He soon found that the liquid food was not disagreeing with him in any way, and knowing so well the necessity of keeping up the strength, he took it in the largest possible quantities, in spite of the loathing it excited.

The following day the symptoms were somewhat abated, and duty seemed to demand that a journey of four or five miles should be taken The result was exactly what might have been anticipated. A relapse took place, bronchitis of the larger tubes set in, severe rigors, with a heightened temperature, were experienced, and every symptom was intensified. The frontal headache, now aggravated by a harassing cough, became exceedingly painful and distressing. The back, across the loins, seemed as if it would break in two. The hip joints, knees, and shoulders also felt as if they were breaking, and as though it would be a relief if they did. The temperature after the relapse remained high for about four days, and all the other symptoms showed little change. At the end of that time, the fever, the headaches, and the other pains gradually abated, leaving the patient more prostrate than ever he had felt in his life up to that time. The abatement of the fever may be considered to have marked the end of the second stage of the disease.

Perhaps the first few days of convalescence after such an attack as has been described constitute the most trying period in the whole course of the malady. Persons who have escaped altogether, or who have had only a slight seizure, do not seem to be able, or even very anxious, to realize what the patient himself feels. They know that very few people actually die of influenza, and therefore they seem to think that "feelings" are of very little account. The patient himself, however, can by no means help taking quite a different view of the situation. No words or metaphors that the writer can think of will convey the least idea to a person who has not had a severe attack, of the profound and hopeless feeling of prostration that follows the subsidence of the fever. The miserable victim feels that he is quite unable either to speak or to think, to be pleased or to be irritable. If he consulted his feelings alone he would say that no power on earth could ever make him either well or happy again. Eating, drinking, and everything else seem labours too great to be entered upon. He finds himself wondering how all the people about him appear to be so absurdly well and strong, and able to do anything they wish to do. As for himself, he thinks, or rather, feels, that if he could heave one sigh and then sink for ever into sleep and forgetfulness he would be happy.

What was the line of treatment adopted by this medical patient in his own case? Salicin, ammoniated tincture of quinine, and other drugs, he found of comparatively little use. As much food as he could take, first fluid, and as soon as possible solid, gave both strength and comfort. Stimulants in the form of champagne, whiskey and water, and ammonia during the height of the fever, and afterwards rich and mature port wine in considerable quantities, and taken with a little solid food every two or three hours, proved to be invaluable. Although the particular person under consideration is ordinarily all but a total abstainer he took almost a whole bottle of port wine daily for seven or eight days during convalescence. To his own decided surprise the wine seemed to promote appetite, help digestion, quicken the activity of the liver and bowels, induce sleep, and in every way to be of the greatest possible service both as medicine and as food. These convictions are expressed with some reluctance, in the fear that the expression of them may be abused by persons who are always on the look out for excuses for wicked self-indulgence. But truth is truth, and he who from any motive whatever distorts or consciously suppresses the truth is neither scientific, religious, nor even moral.

We may now repeat the inquiry, as one of the most important questions connected with the influenza epidemic, —What is the cause of it? The writer, looking back over his personal experience, feels convinced that in his own case, at any rate, the causes were several, and were of the simplest and most ordinary kind. He had been a little out of health for some weeks. Circumstances had compelled him to be irregular with his meals. He had frequently been obliged to work for six or even seven hours between lunch and dinner, and part of that time had been spent in railway travelling. Both appetite and digestion had thus become enfeebled. The long-continued damp weather without frosts had charged the air to its utmost capacity with watery vapour. Thus, not only was the surface of the body chilled, and, so to speak, soaked with moisture, but the nose, throat, windpipe, bronchial tubes, and air vesicles were all constantly bathed and soaked in a mixture of air and water. This persistent chilling of the outer and inner skins gradually exhausted first the peripheral and then the central nervous system. Hence the suddenness of the prostrating attack, followed by a few days of what may be called reactionary fever; and then, as the result of these two, the many days of slowly-disappearing prostration and gradual return to the normal production and distribution of nerve energy. The "microbe" theory, in the writer's own case at any rate, is not necessary, and offers no convincing evidence on its own behalf. With regard to prevention, there is little to be said except this—that persons who keep their general health robust, clothe themselves in suitable winter garments, and avoid exposure to chills and damps, will in all probability escape the epidemic altogether.

